# Utilizing deep learning for accurate assessment of aortic valve stenosis: case series for clinical applications

**DOI:** 10.1186/s44348-025-00047-4

**Published:** 2025-04-25

**Authors:** Jiesuck Park, Jiyeon Kim, Jaeik Jeon, Yeonyee E. Yoon

**Affiliations:** 1 Cardiovascular Center, Division of Cardiology, Department of Internal Medicine, Seoul National University Bundang Hospital, Seongnam, Republic of Korea; 2 Department of Internal Medicine, Seoul National University College of Medicine, Seoul, Republic of Korea; 3 Ontact Health Inc, Seoul, Republic of Korea

**Keywords:** Aortic stenosis, Echocardiography, Artificial intelligence, Deep learning

## Background

### Evolution of AI in evaluation of aortic valve stenosis

Artificial intelligence (AI) is playing an increasingly significant role in medical imaging, and echocardiography is no exception. Initially, the focus was primarily on automating manual measurements from echocardiographic videos or images. However, we have now reached a point where almost all measurements can be performed automatically, and even evaluated according to clinical guidelines [1–4]. For instance, diastolic parameters can be measured automatically, allowing the grading of diastolic dysfunction following guidelines [2]. Similarly, AI can automate the measurement of aortic valve stenosis (AS)-related parameters, calculate the aortic valve area (AVA), and determine the AS severity, eliminating the need for manual intervention [4, 5].

However, AI’s capabilities now extend beyond automating measurements; it has evolved into the realm of expert visual analysis. For instance, while the quantitative assessment of AS relies heavily on Doppler measurements, the diagnostic process typically begins when the examiner visually identifies degenerative changes and limited aortic valve (AV) opening during the initial echocardiographic acquisition, such as the parasternal long-axis (PLAX) or parasternal short-axis (PSAX) views. Likewise, AI can also predict AS by analyzing the PLAX or PSAX views, performing a visual evaluation of the AV comparable to that of an expert. Specifically, we previously developed a deep learning (DL)-based algorithm designed not merely to classify significant or severe AS but also to generate a score, the DL index for AS continuum (DLi-ASc), which progressively reflects increasing AS severity [4]. This algorithm has been validated in large-scale studies, including internal and external testing, confirming its diagnostic and prognostic value.

While our prior study focused on technical validation, including methodological development and performance testing across diverse populations, it did not provide concrete examples of how this algorithm might assist clinical decision-making. In the current study, we retrospectively applied DLi-ASc to a series of real-world AS cases to explore its potential clinical utility. Although DLi-ASc was not used in real-time clinical workflows, we aimed to demonstrate how it could assist clinicians—particularly in challenging or discordant scenarios—by prompting further evaluation or clarifying ambiguous findings. Through this case-based approach, we propose potential roles for the algorithm in supporting AS assessment if integrated into routine practice in the future.

### DL-based AS assessment algorithm

We previously developed a DL-based AS assessment algorithm as a part of an AI-enhanced comprehensive AS evaluation system (US feat_valve.ai, Ontact Health Inc) [4]. This algorithm was developed using a large multicenter dataset and validated across internal and two independent external cohorts, demonstrating excellent diagnostic performance (area under the curve: 0.91–0.99 for any AS, 0.95–0.98 for significant AS, and 0.97–0.99 for severe AS) and independent prognostic value in predicting cardiovascular events. The algorithm employs a 3-dimensional convolutional neural network (r2plus1d18) to analyze transthoracic echocardiography (TTE) video data from PLAX and/or PSAX at the AV level and generate a DLi-ASc (range, 0–100).

**Fig. 1:**
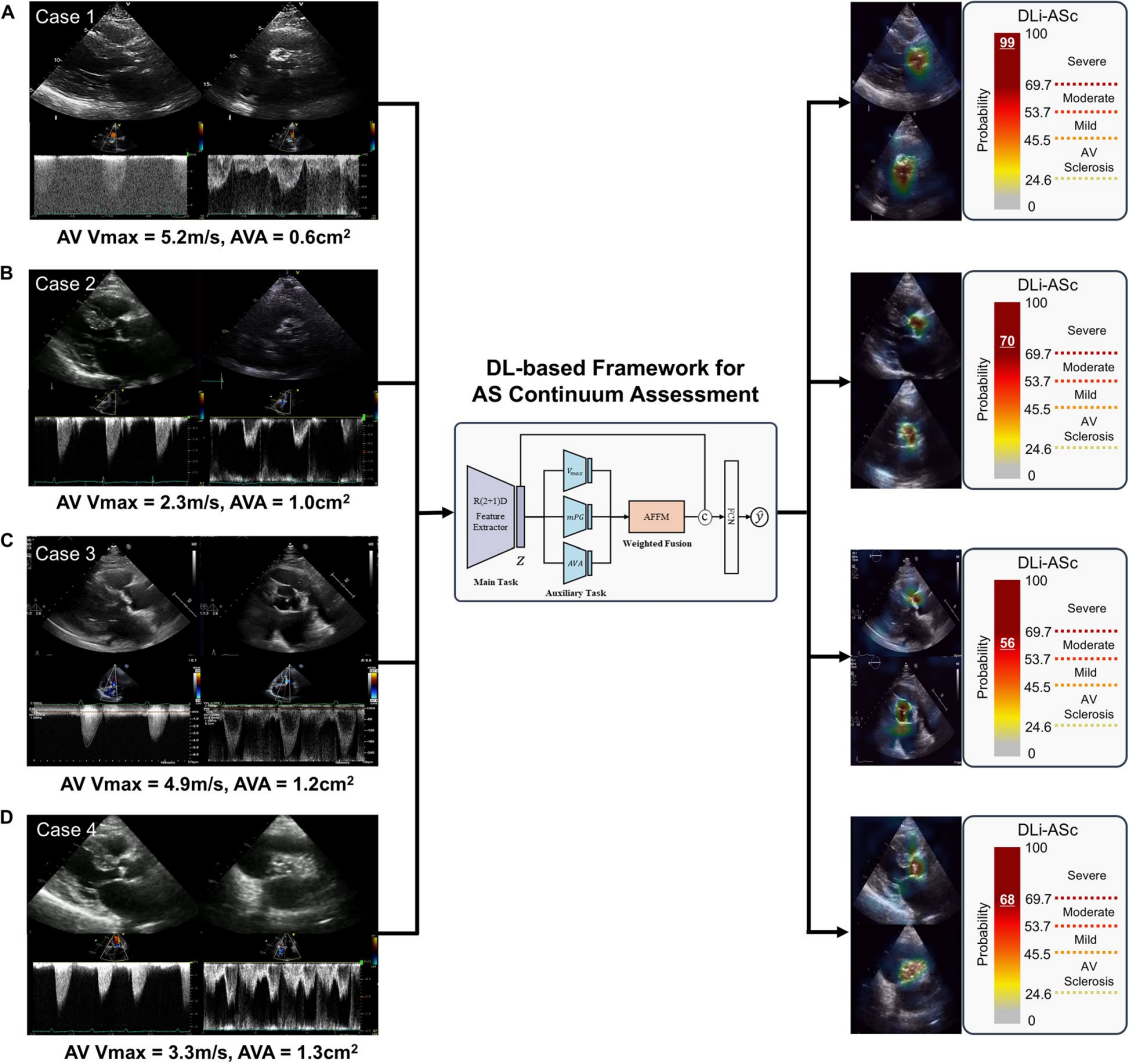
Case series of deep learning (DL)-based framework for enhanced aortic valve stenosis (AS) evaluation via novel DL index for aortic valve stenosis continuum (DLi-ASc). AFFM, adaptive feature fusion module; AV, aortic valve; AVA, aortic valve area; FCN, fully connected network; mPG, mean pressure gradient; V_max_, maximal velocity

Unlike traditional Doppler-based AS severity assessment, which relies directly on flow dynamics, DLi-ASc does not use direct Doppler input. However, it is fundamentally trained to predict Doppler-based AS parameters, leveraging this knowledge to estimate AS severity purely from 2D TTE videos. Our approach captures the continuous progression of AS severity through two strategies: (1) continuous mapping with ordered labels, ensuring that predictions reflect AS as a continuum rather than discrete categories; and (2) multitask learning, where auxiliary tasks involve predicting Doppler-derived quantitative AS parameters, including AV maximal velocity (V_max_), mean pressure gradient (mPG), and AVA. This enhances the model’s ability to differentiate AS severity based on indirect visual cues from TTE videos. The cutoffs for each AS stage, derived from prior analysis, are as follows: 24.6 for AV sclerosis, 45.4 for mild AS, 53.7 for moderate AS, and 69.7 for severe AS (Fig. 1).

## Case reports

To explore the potential clinical utility of DLi-ASc, we retrospectively selected representative AS cases, including both concordant and discordant scenarios. While not randomly chosen, these cases illustrate how DLi-ASc may complement conventional assessment in a variety of clinical contexts. A summary of the baseline clinical characteristics, conventional TTE parameters for AS, and DLi-ASc for each patient is presented below.

### Concordant severe AS: reinforcing early recognition with DLi-ASc

An 86-year-old male patient with hypertension, diabetes, and dyslipidemia presented with worsening dyspnea. TTE revealed a left ventricular (LV) cavity size of 45 mm, normal LV ejection fraction (LVEF) of 59%, and findings consistent with severe AS: V_max_ of 5.2 m/sec, mPG of 67 mmHg, and AVA of 0.6 cm^2^. All conventional Doppler parameters consistently indicated severe AS. The DLi-ASc, derived from PLAX and PSAX images, was 99, further supporting the diagnosis of severe AS (Fig. 1A). Initially planned for transcatheter AV replacement (TAVR), the patient underwent surgical AV replacement (SAVR) due to significant coronary calcification and the risk of coronary obstruction. Postoperatively, the patient showed clinical improvement and remains under outpatient follow-up.

This case illustrates how DLi-ASc may serve as an additional AI-derived assessment tool, particularly in settings where expertise varies, reinforcing the recognition of severe AS. While conventional Doppler parameters provided a definite diagnosis, obtaining DLi-ASc early during TTE image acquisition may help in the early identification of high-risk AS cases and support workflow efficiency, particularly for less experienced operators.

### Paradoxical low-flow, low-gradient AS: unraveling discordance in AS severity assessment

An 83-year-old female patient with no significant cardiovascular risk factors underwent a TTE for the evaluation of dyspnea during hospitalization for pneumonia. The TTE findings revealed a small LV cavity (end-diastolic dimension [EDD] of 37 mm) and a reduced stroke volume index (SVI) of 34 mL/m^2^ despite a preserved LVEF of 62%. Doppler-based AV flow parameters suggested nonsevere AS (AV V_max_, 2.3 m/sec; mPG, 11 mmHg), but the AVA calculated using the continuity equation (CE) was 1.0 cm^2^ (Fig. 1B). Ultimately, the patient was diagnosed with moderate AS, as no additional testing was performed to evaluate the discordance further. During follow-up, the patient experienced recurrent heart failure requiring hospitalization; however, repeat TTE continued to show persistent discordance in AS parameters, and she remained on medical therapy for moderate AS.

A retrospective analysis using our DL system yielded a DLi-ASc of 70, which is near or slightly above the predefined cutoff for severe AS. This case highlights how DLi-ASc may serve as a complementary tool in identifying potentially underestimated AS severity, particularly in paradoxical low-flow low-gradient AS, when conventional TTE findings are inconclusive. While DLi-ASc alone should not guide clinical decisions, a high score in such discordant cases may increase clinical suspicion and support the need for further evaluation—for example, computed tomography-based calcium scoring or follow-up echocardiography. This case illustrates the potential role of DLi-ASc as a supportive indicator that can prompt additional investigation in borderline scenarios.

### Overestimation of AS severity due to LVOT obstruction: a case for careful reassessment

A 69-year-old female patient with diabetes presented with dyspnea and was referred to our center with a diagnosis of severe AS based on an initial TTE, which led to a recommendation for SAVR. Upon repeating TTE at our center, Doppler parameter showed the AV V_max_ of 4.9 m/sec, and the mPG of 45 mmHg; however, the AVA, calculated using the CE, was 1.7 cm^2^ (Fig. 1C). Further evaluation revealed basal septal wall hypertrophy and systolic anterior motion of the mitral valve, leading to dynamic LV outflow tract (LVOT) obstruction. Valsalva maneuver demonstrated an LVOT pressure gradient (PG) of 71 mmHg, confirming significant obstruction. Additionally, the Doppler velocity index (DVI) was 0.53, supporting the elevated AV gradients were primarily due to the LVOT obstruction rather than true severe AS. Based on these findings, the patient’s AS severity was downgraded to moderate.

Notably, the DLi-ASc from the TTE was 56, aligning with moderate AS rather than severe AS. The patient was managed conservatively without surgical intervention and initiated on β-blocker therapy to alleviate the LVOT obstruction. At the 1-year follow-up, the patient’s dyspnea had significantly improved, and repeat TTE showed a reduction in AV Vmax to 4.0 m/sec and mPG to 42 mmHg, with an AVA of 1.5 cm^2^. The LVOT obstruction had also improved, with a PG during the Valsalva maneuver of 44 mmHg.

This case illustrates the potential for overestimating AS severity in the presence of LVOT obstruction, particularly when relying solely on a high Doppler gradient. In such cases, additional assessments, such as DVI and Valsalva maneuver, are essential to differentiate true AS from secondary pressure elevation. While DLi-ASc alone does not directly diagnose LVOT obstruction, it was designed to estimate AS severity based on AV morphology and motion observed in 2D B-mode TTE views (PLAX and/or PSAX), without using direct Doppler input. Its alignment with moderate AS in this case likely reflects this design, in which the AV morphology did not support severe AS despite elevated gradients. As such, DLi-ASc may serve as useful visual consistency check that support further hemodynamic evaluation before confirming severe AS and proceeding with surgical treatment.

### Underestimation of AS severity during atrial fibrillation: a case for reassessment in tachycardia

An 85-year-old female patient with hypertension, diabetes, and atrial fibrillation (AF) underwent TTE as part of her baseline evaluation. The TTE, while the patient was in AF with a heart rate of 100 to 110 beats per minute (bpm), revealed a small LV cavity (EDD of 32 mm) and a preserved LVEF of 69% (SVI of 28 mL/m^2^). The AV V_max_ was 3.3 m/sec, mPG was 25 mmHg, and AVA by CE was 1.3 cm^2^. Based on these findings, the patient was diagnosed with moderate AS without notable difficulty (Fig. 1D). However, the DLi-ASc derived from TTE was 68, a value approaching the severe AS threshold.

Two months later, the patient presented with sudden-onset dyspnea and chest pain, requiring hospitalization. Repeat TTE during readmission, now performed in sinus rhythm with a heart rate of 60 bpm, revealed concordant findings of severe AS, including V_max_ of 4.2 m/sec, mPG of 40 mmHg, and an AVA of 0.9 cm^2^. The patient subsequently underwent TAVR, resulting in significant clinical improvement.

This case highlights the potential for underestimation of AS severity in the presence of AF with tachycardia. Rapid heart rates in AF can lead to reduced stroke volume and cycle length variability, affecting AVA calculation and Doppler-based AS severity assessment. In such cases, reassessment following heart rate control is critical to ensure an accurate diagnosis.

Although the DLi-ASc alone does not diagnose severe AS, its discordant value of 68—approaching the predefined severe AS threshold of 69.7—raises clinical suspicion, especially in the setting of AF and tachycardia. Given that the threshold values were empirically derived and intended to reflect the disease continuum rather than serve as absolute diagnostic cutoffs, borderline scores such as this should be interpreted with caution and in clinical context. In retrospect, the elevated DLi-ASc in this case may suggest that the severity of AS was underestimated during the initial evaluation, highlighting its potential role as a supportive tool in complex hemodynamic conditions. A strategy incorporating repeat TTE after heart rate control or additional imaging (e.g., transesophageal echocardiography, AV calcium scoring) may be beneficial in such scenarios to ensure appropriate clinical management.

## Discussion

AI advancements are driving significant change in echocardiographic analysis, with most manual measurements now capable of being automated. Beyond mere automation, AI has evolved to emulate expert visual analysis, reshaping how echocardiographic data is interpreted. DLi-ASc represents an AI-driven approach to AS severity assessment, leveraging DL to analyze AV morphology and motion from 2D TTE videos. Unlike traditional methods that rely on Doppler-derived hemodynamic parameters, DLi-ASc provides a complementary, continuum-based assessment that may aid in identifying discordance in AS evaluation. It is important to note that this study was not intended as a new validation of the DLi-ASc algorithm, but rather as a case-based exploration of its potential clinical utility following prior large-scale validation [4].

Through the presented cases, we explored the potential clinical applications of DLi-ASc: (1) serving as an initial indicator that may raise suspicion for AS, potentially prompting further Doppler-based assessment when significant AS is suggested; and (2) providing complementary information in cases with discordant or inconclusive findings, helping to flag inconsistencies that may indicate potential measurement errors or the need for reassessment. This application is particularly relevant in cases where traditional assessment is challenging due to low-flow conditions, dynamic LVOT obstruction, or hemodynamic variability in AF. While DL-ASc does not replace Doppler-based assessment, it may function as a visual aid, enhancing clinician awareness and encouraging further evaluation when AS severity is uncertain.

### Limitations

As an exploratory case series, this study is limited in scope. Moreover, like other image-based AI tools, the performance of DLi-ASc may be affected by suboptimal image quality, acoustic window limitations, or coexisting valvular pathologies. These potential sources of error should be considered when interpreting DLi-ASc in clinical practice.

### Future perspectives

To fully integrate DLi-ASc into clinical workflow, further prospective validation in diverse clinical settings is required. This includes assessing its performance in primary care centers and resource-limited environments where expert echocardiographic interpretation may not be readily available. Additionally, the potential for real-time integration into echocardiographic workflow should be explored. Although DLi-ASc is currently applied to post-exam, its architecture allows for real-time implementation, which could aid less experienced examiners by providing immediate AS severity assessment and guiding additional quantitative evaluations. Furthermore, ongoing research should focus on identifying factors that influence DLi-ASc predictions, such as valve echogenicity, motion dynamics, and image quality. The incorporation of explainable AI techniques, as demonstrated in the provided saliency maps (Fig. 1), enhances the interpretability of AI-based assessment, allowing clinicians to better understand how the model prioritizes echocardiographic features. These advancements will be essential for refining the robustness and clinical acceptance of AI-driven tools in echocardiography. At the same time, broader challenges inherent to AI development—including training bias, dependence on expert-derived labels, variable image quality, and limited generalizability to untrained pathophysiologies—must be acknowledged and addressed to ensure responsible clinical adoption.

## Conclusions

DLi-ASc represents a novel AI-based approach to AS severity assessment, bridging the gap between automated quantification and expert visual analysis. Providing a continuum-based assessment may aid in screening for AS and identifying discordant cases, supporting more informed clinical decision-making. However, DLi-ASc should be viewed as a complementary tool rather than a standalone diagnostic method. Further validation is necessary to establish its clinical role and reliability across diverse patient populations. With ongoing refinements and integration into echocardiographic workflows, DLi-ASc has the potential to enhance the accuracy, efficiency, and consistency of AS evaluation, ultimately improving patient outcomes.

## Data Availability

Please contact the corresponding authors to request the minimal anonymized dataset.

## Abbreviations

AF: Atrial fibrillation
AI: Artificial intelligence
AS: Aortic valve stenosis
AV: Aortic valve
AVA: Aortic valve area
bpm: Beats per minute
CE: Continuity equation
DL: Deep learning
DLi-ASc: Deep learning index for aortic valve stenosis continuum
DVI: Doppler velocity index
EDD: End-diastolic dimension
LV: Left ventricular
LVEF: Left ventricular ejection fraction
LVOT: Left ventricular outflow tract
mPG: Mean pressure gradient
PG: Pressure gradient
PLAX: Parasternal long-axis
PSAX: Parasternal short-axis
SAVR: Surgical aortic valve replacement
SVI: Stroke volume index
TAVR: Transcatheter aortic valve replacement
TTE: Transthoracic echocardiography
V_max_: Maximal velocity
